# Precipitous changes in nomenclature and definitions—NAFLD becomes SLD: Implications for and expectations of AASLD journals

**DOI:** 10.1097/HC9.0000000000000318

**Published:** 2023-11-09

**Authors:** Harmeet Malhi, Robert S. Brown, Joseph K. Lim, Nancy Reau, Elliot B. Tapper, Carmen Chak-Lui Wong, Gregory J. Gores

**Affiliations:** 1 Division of Gastroenterology and Hepatology, Mayo Clinic, Rochester, Minnesota, USA; 2 Division of Gastroenterology and Hepatology, Weill Cornell Medical College, New York, New York, USA; 3 Yale Viral Hepatitis Program, Yale University School of Medicine, New Haven, Connecticut, USA; 4 Section of Hepatology, Division of Digestive Diseases, Department of Internal Medicine, Rush University Medical Center, Chicago, Illinois, USA; 5 Division of Gastroenterology and Hepatology, University of Michigan, Ann Arbor, Minnesota, USA; 6 Department of Pathology, The University of Hong Kong, Hong Kong, China; 7 State Key Laboratory of Liver Research, The University of Hong Kong, Hong Kong, China

Recently a multi-stakeholder effort by the American Association for the Study of Liver Diseases (AASLD), the European Association for the Study of Liver Diseases (EASL), in collaboration with the Association Latinoamericana para el Estudio del Higado (ALEH) with other stakeholders developed a consensus on a change in nomenclature and diagnostic criteria for NAFLD and NASH. The rationale was to remove potentially stigmatizing language from the terms and a reliance on exclusionary confounding definitions.^1^ Steatotic liver disease (SLD) was chosen as an umbrella term with subcategories. Metabolic dysfunction–associated steatotic liver disease (MASLD) was selected for those patients with SLD and a cardiometabolic risk factor, and those without cardiometabolic risk factors are referred to as cryptogenic SLD or possible MASLD. Metabolic dysfunction–associated steatohepatitis (MASH) replaced NASH. A new category was created for those with “MASLD” who consume moderate amounts of alcohol (20-50 g/day for women and 30-60 g/day for men), Met-ALD. Alcohol-associated liver disease (>50/60 g/day for women and men), irrespective of the presence of cardiometabolic risk factors, is designated as ALD. Definitions and a calculator for classifying patients are available on the AASLD website (https://www.aasld.org/new-nafld-nomenclature).

What are the implications of these changes in nomenclature and definitions for authors and the AASLD journals (*Clinical Liver Disease*, HEPATOLOGY, *Hepatology Communications*, and *Liver Transplantation*)? The overarching mission of the societal suite of journals is to serve as a platform for disseminating new and impactful research findings to a multi-stakeholder community and the public. Agreed-upon definitions are requisite for communicating the knowledge and for making comparisons between studies over time. Moreover, the agreed-upon nomenclature and definitions are also critical for developing health policy recommendations and for regulatory agency approval of therapeutic agents, biomarkers, and medical devices. Therefore, it is incumbent for AASLD journals to adopt these changes with alacrity. The adaptation of these changes in nomenclature and definitions is more critical for human studies, given the complexity, heterogeneity, and known confounders inherent to human studies. In animal models, the terminology should be consistent with the human nomenclature. Given the highly controlled methodological conditions, the exact application of the definitions of human disease is contextually relevant. For example, defined alcohol intake in a diet-induced obesity murine model of Met-ALD or defining a dietary model of MASH in contrast with steatotic liver disease (without features of cardiometabolic risk factors).

The editorial teams and AASLD representatives have agreed on the following transition to implementing this change.

## HUMAN STUDIES

- Manuscripts in press or currently under review will not be required to adopt the new terminology and definitions.
- New manuscript submissions should employ the revised terminology and definitions going forward. If the appropriate terminology is not used during the initial or subsequent manuscript revision, articles will not be viewed as acceptable for publication unless the change to the new terms is made.

## PRECLINICAL STUDIES

- Preclinical studies submitted will be reviewed without an insistence they use the new nomenclature. If a resubmission of the revised manuscript is encouraged, the nomenclature can be adopted in the revised manuscript. Preclinical studies may not have sufficient documentation of cardiometabolic risk factors to warrant the designation MASLD or MASH. For such studies, the term diet-induced steatohepatitis with fibrosis could be used. Those studies without inflammation and fibrosis would fall under the SLD category.

We anticipate that by mid-2024, all human studies on SLD published in AASLD journals will adhere to the new specified nomenclature and definitions. We appreciate that for many ongoing studies, this will require revised assessment and stratification of patient populations and data; however, if these changes are to be meaningful and impactful, they need to be regarded seriously and adopted now!
